# Protocol for generating transgene-free naive human induced pluripotent stem cells from somatic cells using modified Sendai viral system

**DOI:** 10.1016/j.xpro.2025.103700

**Published:** 2025-03-22

**Authors:** Kaho Washizu, Shinya Yamanaka, Akira Kunitomi

**Affiliations:** 1 Gladstone Institute of Cardiovascular Disease, San Francisco, CA 94158, USA; 2 Center for iPS Cell Research and Application (CiRA), Kyoto University, Kyoto 606-8507, Japan; 3 CiRA Foundation, Kyoto 606-8397, Japan; 4 Department of Anatomy, University of California, San Francisco, San Francisco, CA 94143, USA

**Keywords:** Cell biology, Cell culture, Stem cells, Biotechnology and bioengineering

## Abstract

Sendai virus (SeV) vector represents a powerful tool for generating naive and primed human induced pluripotent stem cells (iPSCs) from somatic cells. Here, we present a protocol for the generation of transgene-free naive human iPSCs from human dermal fibroblasts (HDFs) and human peripheral mononuclear cells (PBMCs) using a modified SeV vector system. We describe steps for thawing the HDFs or PBMCs, reseeding HDFs, SeV vector infection, reseeding the SeV-infected HDFs or PBMCs on an irradiated mouse embryonic fibroblast (iMEF) plate, switching to t2iLGö+Y medium, passaging the generated naive iPSCs, removing the SeV vectors, and cryopreserving the naive iPSCs.

For complete details on the use and execution of this protocol, please refer to Kunitomi et al.^1^^,^^2^

## Highlights

• Derivation of transgene-free naive human induced pluripotent stem cells (iPSCs)
• Technique for generating naive human iPSCs from human dermal fibroblasts (HDFs)
• Generation of naive human iPSCs from human peripheral blood mononuclear cells (PBMCs)
• Protocol for converting HDF-derived naive iPSCs to feeder-free culture conditions

## Abstract (editor highlights)

Publisher’s note: Undertaking any experimental protocol requires adherence to local institutional guidelines for laboratory safety and ethics.

## Graphical abstract

**Figure:**
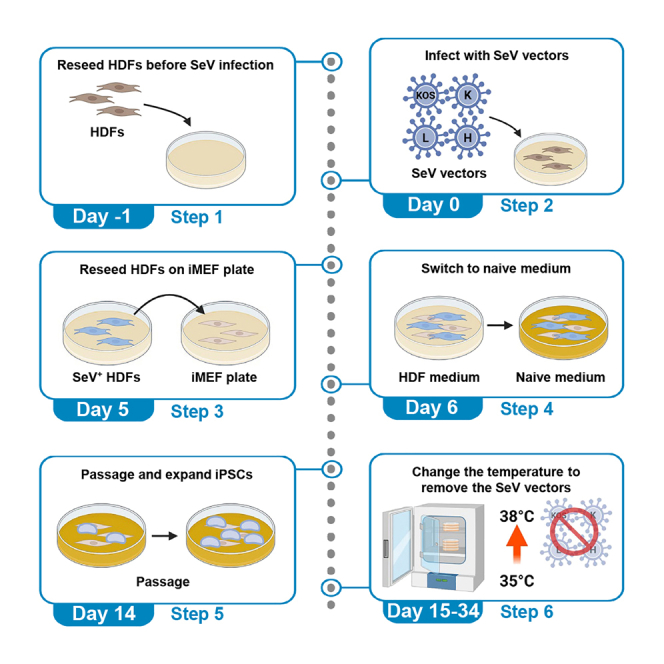

## Before you begin

Here, we describe a stepwise method for the simple and rapid generation of transgene-free, functional naive human iPSCs using modified SeV vectors. This method enables the generation of naive human iPSCs not only from HDFs but also from PBMCs by using *LMYC* in place of *CMYC*. Furthermore, structural modifications to the SeV vector allow for its removal simply by adjusting the culture temperature.^1^ As an additional option, we introduce the use of *H1FOO-DD* as a reprogramming factor to generate higher-quality naive human iPSCs^2^ and the method for converting HDF-derived naive human iPSCs to feeder-free culture. This method does not require specialized or advanced techniques beyond those of conventional SeV vector-based human iPSC generation protocols.

### Medium and incubator setup

**Timing:** 1 h

Prepare the media before starting the experiment. The prepared media can be stored at 4°C for up to 2 weeks. However, if only a small amount of medium is required, it can be aliquoted into 50 mL tubes and stored at −20°C for up to 6 months.

It is highly recommended to calibrate the temperature of the incubator using a high-precision thermometer before starting the experiment. Temperature errors have a significant impact on the results of experiments.

1. Fibroblast medium preparation: Dulbecco’s modified Eagle medium (DMEM) supplemented with 10% fetal bovine serum (FBS). This medium can also be used for irradiated mouse embryonic fibroblast (iMEF) culture.
2. PBMC medium preparation: StemSpan-AOF with recombinant human SCF, TPO, Flt3, IL-6, and IL-3.
3. t2iLGö^3^+Y medium preparation: NDiff 227 with CHIR99021, PD0325901, human LIF, Gö6983, and Y-27632.

***Note:*** Add Y-27632 to t2iLGö+Y medium only just before use.

4. Wash medium preparation: DMEM/F-12, GlutaMAX supplement with 0.1% bovine albumin fraction (BSA).

## Key resources table

**Table:** 

REAGENT or RESOURCE	SOURCE	IDENTIFIER
**Bacterial and virus strains**		

SeV(PM)KOS/TS12ΔF: KOS vector	ID Pharma	Contact akira.kunitomi@gladstone.ucsf.edu or ID Pharma (web-info@iromgp.com)
SeV18+KLF4/TS12ΔF: KLF4 vector	ID Pharma	Contact akira.kunitomi@gladstone.ucsf.edu or ID Pharma (web-info@iromgp.com)
SeV(HNL)L-MYC/TS15ΔF: LMYC vector	ID Pharma	Contact akira.kunitomi@gladstone.ucsf.edu or ID Pharma (web-info@iromgp.com)
SeV18 + H1FOO-DD/TS15ΔF: H1FOO-DD vector	ID Pharma	Contact akira.kunitomi@gladstone.ucsf.edu or ID Pharma (web-info@iromgp.com)

**Chemicals, peptides, and recombinant proteins**		

DMEM (4.5 g/L glucose) with L-Gln, without sodium pyruvate, liquid	Nacalai Tesque	Cat# 08459-64
Fetal bovine serum, heat inactivated	Sigma-Aldrich	Cat# F4135-500ML
Gelatin	STEMCELL Technologies	Cat# 07903
D-PBS(−)	Gibco	Cat# 14190144
TrypLE select	Gibco	Cat# 12563029
StemSpan-AOF (Successor to StemSpan-ACF)	STEMCELL Technologies	Cat# 100-0130
Recombinant human SCF protein	R&D Systems	Cat# 255-SC
Recombinant human thrombopoietin Protein	R&D Systems	Cat# 288-TPN
Recombinant human Flt-3 ligand/FLT3L protein	R&D Systems	Cat# 308-FK
Recombinant human IL-6 protein	R&D Systems	Cat# 206-IL
Recombinant human IL-3 protein	R&D Systems	Cat# 203-IL
DMEM/F-12, GlutaMAX supplement	Gibco	Cat# 10565018
Bovine albumin fraction V (7.5% solution)	Gibco	Cat# 15260037
NDiff227	Takara Bio	Cat# Y40002
CHIR99021	Sigma-Aldrich	Cat# SML1046
PD0325901	Sigma-Aldrich	Cat# PZ0162
Gö6983	Sigma-Aldrich	Cat# G1918
Recombinant human LIF	PeproTech	Cat# 300-05
CultureSure Y-27632	FUJIFILM Wako	Cat# 036-24023
Accutase	Innovative Cell Technologies	Cat# AT104
STEM-CELLBANKER	Takara Bio	Cat# 11924
Matrigel ESC-qualified matrix	Corning	Cat# CLS354277

**Critical commercial assays**		

RNeasy Mini Kit	QIAGEN	Cat# 74104
PrimeScript RT Master Mix	Takara Bio	Cat# RR036A
TaqMan Fast Advanced Master Mix for qPCR	Thermo Fisher Scientific	Cat# 4444557

**Experimental models: Cell lines**		

Human dermal fibroblast: TIG113 (21yo, female)	JCRB Cell Bank	ID: JCRB0539
Human dermal fibroblast: TIG120 (6yo, female)	JCRB Cell Bank	ID: JCRB0542
Uncharacterized cryopreserved human PBMC	Cellular Technology Limited	Cat# CTL-UP1
CF1 mouse embryonic fibroblasts, irradiated (iMEF feeder cells, 2 × 10^6^ or 4 × 10^6^ cells/vial)	Thermo Fisher Scientific	Cat# A34181

**Oligonucleotides**		

SEV (Mr04269880_mr)	Thermo Fisher Scientific	Cat# 4331182
GAPDH (Hs02786624_g1)	Thermo Fisher Scientific	Cat# 4331182

**Other**		

Cell-IQ Stackable multigas CO_2_/O_2_ incubator	PHCBi	Cat# MCO-50M-PA
Countess 3 automated cell counter	Thermo Fisher Scientific	Cat# A49862
Countess cell counting chamber slides	Thermo Fisher Scientific	Cat# C10283
Trypan blue stain 0.4%	Thermo Fisher Scientific	Cat# T10282
Millex-HP syringe filter unit, 0.45 μm	Merck	Cat# SLHPR33RB
MicroAmp Fast 96-well reaction plate (0.1 mL)	Thermo Fisher Scientific	Cat# 4346907
MicroAmp optical adhesive film	Thermo Fisher Scientific	Cat# 4311971

## Materials and equipment

**Table:** Fibroblast medium

Reagent	Final concentration	Amount
DMEM (4.5 g/L Glucose) with L-Gln, without Sodium Pyruvate, liquid	–	500 mL
FBS	10%	56 mL
Total	–	556 mL

Store at 4°C for up to 2 weeks.

**Table:** PBMC medium (for non-T cells)

Reagent	Stock concentration	Final concentration	Amount
StemSpan-AOF	–	–	50 mL
Recombinant Human SCF Protein∗	100 μg/mL	100 ng/mL	50 μL
Recombinant Human Thrombopoietin Protein∗	100 μg/mL	100 ng/mL	50 μL
Recombinant Human Flt-3 Ligand/FLT3L Protein∗	100 μg/mL	100 ng/mL	50 μL
Recombinant Human IL-6 Protein∗	100 μg/mL	50 ng/mL	25 μL
Recombinant Human IL-3 Protein∗	100 μg/mL	20 ng/mL	10 μL
Total	–	–	50 mL

∗Resuspend with 0.1% BSA-containing PBS

Make 50 mL tube aliquots and store them at −20°C. Store at 4°C for up to 2 weeks after thawing.

**Table:** t2iLGö+Y medium for naive iPSC culture

Reagent	Final concentration	Amount
NDiff 227	–	50 mL
CHIR99021 (10 mM)	1 μM	5 μL
PD0325901 (10 mM)	1 μM	5 μL
Recombinant Human LIF (10 μg/mL)	10 ng/mL	50 μL
Gö6983 (5 mM)	2.5 μM	25 μL
Y-27632 (10 mM)	10 μM	5 μL
Total	–	50 mL

Add Y-27632 only just before use.

Make 50 mL tube aliquots and store them at −20°C. Store at 4°C for up to 2 weeks after thawing.

**Table:** Wash medium for naive iPSC passage

Reagent	Final concentration	Amount
DMEM/F-12, GlutaMAX supplement	–	500 mL
Bovine Albumin Fraction V (7.5% solution)	0.1%	6.7 mL
Total	–	506.7 mL

Store at 4°C for up to 2 weeks.

***Alternatives:*** Except for the SeV vectors, the reagents, cells, and equipment required for this method can be substituted with alternatives that have equivalent components, functions, or characteristics; however, the use of materials different from those introduced here could require further optimization.

## Step-by-step method details

### Reprogram HDFs or PBMCs

**Timing: 34 days**

This section introduces a method for generating naive human iPSCs from HDFs or PBMCs. About the reprogram HDFs, it provides a step-by-step description, starting with the thawing of frozen HDF stocks, infection with the SeV vectors, preparation of iMEF plates, seeding of SeV-infected HDFs onto these plates, passaging after the appearance of naive iPSC colonies, and temperature adjustments. The process concludes with confirmation of SeV genome removal and cryopreservation of the established iPSC clones.

**Figure 1:**
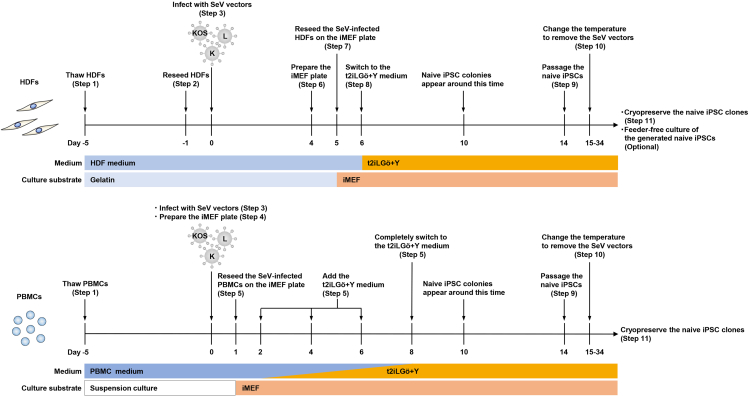
Experimental design for the derivation of naive human iPSCs from HDFs or PBMCs

***Note:*** It is not recommended to use the iMEF plates two days or more after the seeding. If it is to be used on the second day or later, change the medium with the fibroblast medium the day after re-seeding and every other day thereafter.

Regarding the reprogram from PBMCs, the overall workflow is similar to that for HDFs; however, key differences include the use of suspension culture up to the point of SeV infection, the seeding of SeV-infected cells onto iMEF plates on day 1, and the gradual transition of the medium from PBMC medium to t2iLGö+Y (Figure 1).

***Note:*** It is possible to cryopreserve cells and resume experiments by thawing them on any day during this process. However, cryopreservation before day 14 may cause reduction of reprogramming efficiency and should only be considered in emergencies.

**Figure 2:**
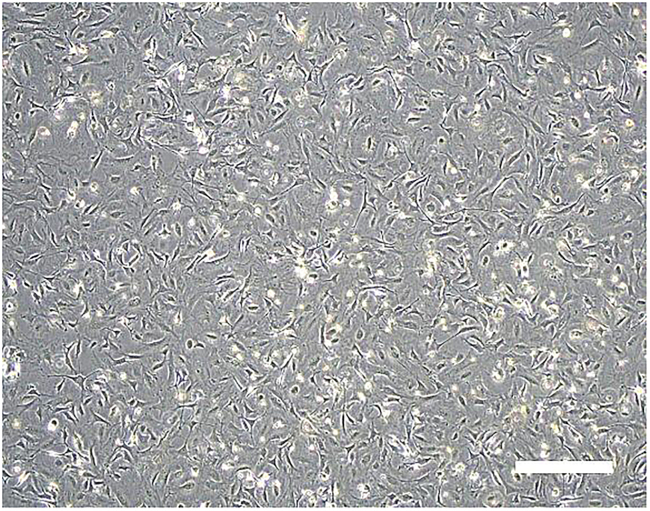
iMEF feeder cells Representative phase-contrast images of iMEF feeder cells seeded in a 6-well plate. Scale bar, 200 mm.

***Note:*** Mitotically inactivated MEFs can also be used.

***Note:*** The iMEF plate can be used from as early as 3 h after the iMEF seeding. However, it is highly recommended to use the iMEF plate after iMEFs have been incubated for more than 12 h.

1. Thawing and culturing of HDFs or PBMCs for reprogramming (Day −5).
  **HDF protocol** (Timing: 1–2 h)
  a. Warm the fibroblast medium to 37°C.
  b. Add 0.1% gelatin solution to a 10 cm dish and incubate at 20°C–25°C for at least 15 min.
  c. Take a frozen HDF vial from a liquid nitrogen (LN2) tank.
  d. Thaw the frozen HDF vial in a 37°C water bath.
  e. Transfer the content of the vial into a 15 mL tube containing 5 mL of the HDF medium.
  f. Centrifuge the tube at 500 × *g* for 5 min.
  g. Aspirate the supernatant.
  h. Resuspend the cell pellet in 10 mL of the fibroblast medium.
  i. Aspirate the gelatin solution from the 10 cm dish just before the cell plating.
  j. Plate the cell suspension to the gelatin-coated 10 cm dish (5 × 10^5^ cells / dish).
  k. Incubate the cells at 37°C with 5% CO_2_, and change the medium every other day.
  **PBMC protocol** (Timing: 1–2 h)
  ***Note:*** This protocol selects non-T cell mononuclear cells for reprogramming from PBMCs during the first 5 days of culture.
  l. Warm the PBMC medium to 37°C.
  m. Take a frozen PBMC vial from the LN2 tank.
  n. Thaw the frozen PBMC vial in a 37°C water bath.
  o. Transfer the content of the vial into a 15 mL tube containing 5 mL of the PBMC medium.
  p. Centrifuge the tube at 500 × *g* for 5 min.
  q. Aspirate the supernatant.
  r. Resuspend the cell pellet in 1 mL of the PBMC medium.
  s. Count the cell number using an automated cell counter.
  t. Seed the cells at a density of < 3 × 10^6^ cells / 2 mL / well in a 6-well plate.
  u. Incubate at 37°C with 5% CO_2_ for 5 days until SeV vectors infection (no need for medium change).
    **Pause point:** Non-T cell mononuclear cells selected through Steps 1–10 can be cryopreserved at temperatures below −80°C. Upon thawing, the cells can immediately proceed to Step 11 and beyond.
2. Reseed HDFs before SeV vector infection (Day −1, HDF protocol Only) (Timing: 1–2 h).
  a. Warm the fibroblast medium to 37°C.
  b. Add 0.1% gelatin solution to a 6-well plate and incubate at 20°C–25°C for at least 15 min.
  c. Wash HDFs in the 10 cm dish with 10 mL of PBS.
  d. Add 4 mL of TrypLE select.
  e. Incubate at 37°C for 10 min.
  f. Dissociate the cells by pipetting and transfer the cell suspension into a 15 mL tube.
  g. Rinse the dish by adding 10 mL of the fibroblast medium to the dish.
  h. Add the suspension to the same 15 mL tube to collect the cells remaining in the dish.
  i. Centrifuge at 500 × *g* for 5 min.
  j. Aspirate the supernatant.
  k. Resuspend the cell pellet in 1 mL of the fibroblast medium.
  l. Count the cell number using an automated cell counter.
  m. Add the fibroblast medium to a concentration of 1.0 × 10^5^ cells/ml.
  n. Aspirate the gelatin solution from the 6-well plate just before the cell plating.
  o. Plate the cell suspension into the gelatin-coated 6-well plate (2.0 × 10^5^ cells/well) and reseed the cells in an extra well for the next day’s cell count.
  p. Incubate the cells at 37°C with 5% CO_2_.
3. Infect with SeV vectors (Day 0).
  **HDF protocol** (Timing: 2 h)
  a. Set the incubator to 35°C, 5% CO_2_ and 5% O_2_.
  b. Warm the fibroblast medium to 20°C–25°C.
    **CRITICAL:** Do not warm the medium to 37°C.
  c. Detach the cells from the well prepared for cell counting using TrypLE select as described in the steps 14 through 18.
  d. Count the cells to calculate the amount of SeV to be prepared.
  e. Thaw each SeV vector-containing tube by immersing the bottom of the tube in a 37°C water bath, ensuring that the tube is submerged up to the level of the vector solution, for 10 s.
    i. Remove the tube from the water bath.
    ii. Completely thaw the tube on ice.
      ***Note:*** It takes about 30 min for the vector solution to completely thaw.
  f. Prepare 1 mL of the fibroblast medium in a 1.5 mL tube for mixing the following SeV vectors.
    i. Add KOS vector at a MOI of 5 to the tube.
    ii. Add KLF4 vector at a MOI of 5 to the tube.
    iii. Add LMYC vector at a MOI of 5 to the tube.
    iv. (Optional) Add H1FOO-DD vector at a MOI of 0.3 to the tube.
    v. Mix thoroughly by pipetting.
      ***Note:*** KOS vector, KLF4 vector, and LMYC vector are the minimum requirements for naive reprogramming. H1FOO-DD vector is optional but recommended for inclusion in the naive reprogramming cocktail to enhance reprogramming efficiency.^2^
  g. Remove the medium from the HDF-containing well.
  h. Add the SeV vector-containing fibroblast medium into the well.
    **CRITICAL:** Do not add each SeV vector directly to the well one-by-one.
    **CRITICAL:** Use up the vectors after thawing and do not repeat freeze/thaw cycles.
  i. Incubate at 35°C, 5% CO_2_ and 5% O_2_.
  j. After 24 h, remove the medium and add 2 mL of the fibroblast medium.
  k. Change the medium with the fibroblast medium every other day and incubate for 5 days.
    **CRITICAL:** Use the fibroblast medium after it has reached at least 20°C–25°C. Do not use media that has been warmed to 37°C or chilled media that has just been removed from a refrigerator at 4°C.
  **PBMC protocol** (Timing: 1 h)
  l. Set the incubator to 35°C, 5% CO_2,_ and 5% O_2_.
  m. Warm the PBMC medium to 20°C–25°C.
    **CRITICAL:** Do not warm the medium to 37°C.
  n. Collect the cells to a 15 mL tube from the 6-well plate.
  o. Centrifuge the tube at 500 × *g* for 5 min.
  p. Aspirate the supernatant.
  q. Resuspend the cell pellet in 0.5–1 mL of the PBMC medium.
  r. Count the cells to calculate the amount of SeV to be prepared.
  s. Reseed the cells in a 48-well plate (2 × 10^5^ cells / 200 μl / well).
  t. Incubate the cells at 35°C, 5% CO_2,_ and 5% O_2_ until the SeV vectors are thawed.
  u. Thaw each SeV vector-containing tube by immersing the bottom of the tube in a 37°C water bath, ensuring that the tube is submerged up to the level of the vector solution, for 10 s.
    i. Remove the tube from the water bath.
    ii. Completely thaw the tube on ice.
      ***Note:*** It takes about 30 min for the vector solution to completely thaw.
  v. Prepare 1.5 mL tube for mixing the following SeV vectors.
    i. Add KOS vector at a MOI of 5 to the tube.
    ii. Add KLF4 vector at a MOI of 5 to the tube.
    iii. Add LMYC vector at a MOI of 5 to the tube.
    iv. (Optional) Add H1FOO-DD vector at a MOI of 0.3 to the tube.
    v. Mix thoroughly by pipetting.
      ***Note:*** KOS vector, KLF4 vector, and LMYC vector are the minimum requirements for naive reprogramming. H1FOO-DD vector is optional but recommended for inclusion in the naive reprogramming cocktail to enhance reprogramming efficiency.^2^
  w. Add the mixed vector solution to the 48-well plate containing the cells.
  x. Culture the cells in an incubator at 35°C, 5% CO_2_ and 5% O_2_.
    **CRITICAL:** Do not add each SeV vector directly to the well one-by-one.
    **CRITICAL:** Use up the vectors after thawing and do not repeat freeze/thaw cycles.
4. Prepare the iMEF plate (Day 0, PBMC protocol only) (Timing: 1 h).
  a. Warm the fibroblast medium to 37°C.
  b. Add 0.1% gelatin solution to two 6-well plates and incubate at 20°C–25°C for at least 15 min.
  c. Take a frozen iMEF vial (4 × 10^6^ cells/vial) from the LN2 tank. Usually, one vial containing 4 × 10^6^ cells is enough for two 6-well plates.
  d. Thaw the frozen vial of iMEF in a 37°C water bath.
  e. Transfer the content of the vial into a 50 mL tube containing 23 mL of the fibroblast medium.
  f. Aspirate the gelatin solution from the wells just before the cell plating.
  g. Plate the cell suspension to the two gelatin-coated 6-well plates (2.5–3.5 × 10^5^ cells / 2 mL / well).
  h. Incubate at 37°C with 5% CO_2_. (Figure 2).

5. Reseed the SeV-infected PBMCs on the iMEF plate (Day 1, PBMC protocol only) (Timing: 1–2 h).
  a. Warm the PBMC medium to 20°C–25°C.
    **CRITICAL:** Do not warm the medium to 37°C.
  b. Collect the cells from the 48-well plate and place them in a 1.5 mL tube.
  c. Add 1 mL of the PBMC medium to the 48-well plate and collect the remaining cells in the wells.
  d. Centrifuge the tube at 500 × *g* for 5 min.
  e. Aspirate the supernatant.
  f. Resuspend the cells in 1 mL of the PBMC medium.
  g. Count the cell number using an automated cell counter.
  h. Add the PBMC medium to a concentration of 2 × 10^5^ cells/ 1.5 mL.
  i. Wash the 6-well iMEF plate twice with PBS.
  j. Plate the cells on the iMEF plate (2.0 × 10^5^ cells/ 1.5 mL / well).
  k. Incubate at 35°C, 5% CO_2_, and 5% O_2_.
  l. After 2, 4, and 6 days of the SeV vectors infection, add 1.5 mL of t2iLGö+Y medium each time. The amount of the PBMC medium in a well will be 6 mL at day 6 after the SeV infection.
  m. After 8 days of the SeV vectors infection, aspirate entire medium and replace it with 2 mL of t2iLGö+Y medium.
    ***Note:*** Typically, dome-shaped naive iPSC-like colonies with shiny edges appear around 10 days after the SeV infection. If colonies do not appear after 14 days, it is highly likely that the reprogramming was not successful.
6. Prepare the iMEF plate (Day 4, HDF protocol only) (Timing: 1 h).
  a. Warm the fibroblast medium to 37°C.
  b. Add 0.1% gelatin solution to two 6-well plates and incubate at 20°C–25°C for at least 15 min.
  c. Take a frozen iMEF vial (4 × 10^6^ cells/vial) from the LN2 tank. Usually, one vial containing 4 × 10^6^ cells is enough for two 6-well plates.
  d. Thaw the frozen vial of iMEF in a 37°C water bath.
  e. Transfer the content of the vial into a 50 mL tube containing 23 mL of the fibroblast medium.
  f. Aspirate the gelatin solution from the wells just before the cell plating.
  g. Plate the cell suspension to the two gelatin-coated 6-well plates (2.5–3.5 × 10^5^ cells / 2 mL / well).
  h. Incubate at 37°C with 5% CO_2_ (Figure 2).

***Note:*** Mitotically inactivated MEFs can also be used.

***Note:*** The iMEF plate can be used from as early as 3 h after the iMEF seeding. However, it is highly recommended to use the iMEF plate after iMEFs have been incubated for more than 12 h.

***Note:*** It is not recommended to use the iMEF plates two days or more after the seeding. If it is to be used on the second day or later, change the medium with the fibroblast medium the day after re-seeding and every other day thereafter.

**Figure 3:**
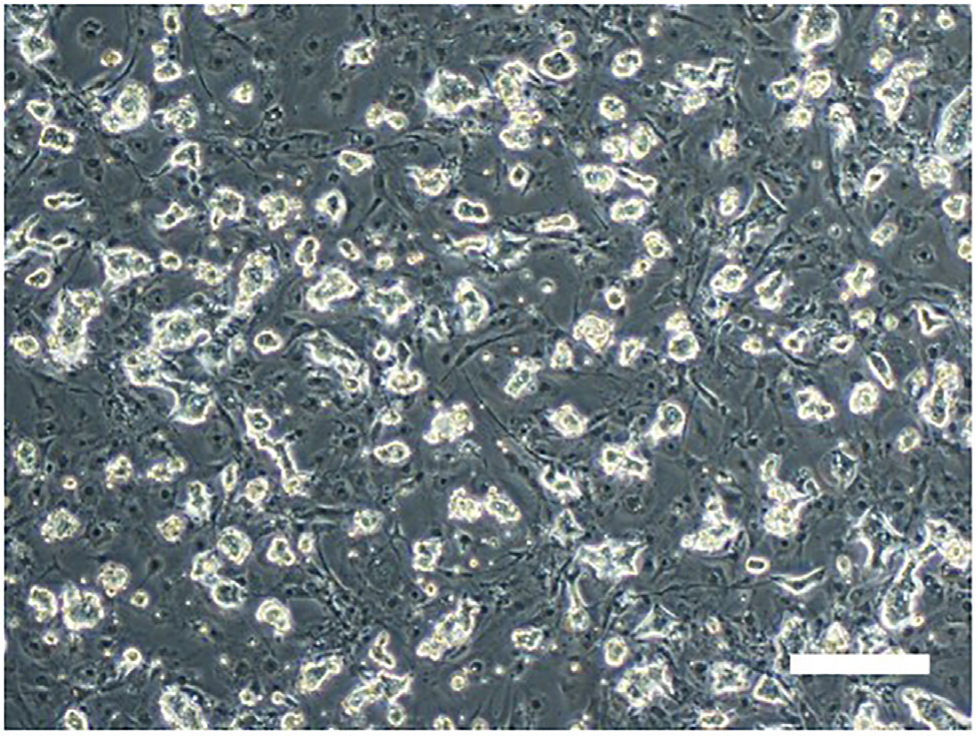
Naive iPSC colonies derived from HDFs Representative phase-contrast images of naive iPSCs reprogrammed with naive reprogramming cocktail (SeV-KOS, SeV-KLF4/TS12, and SeV-LMYC vectors) on iMEF feeder cells. Scale bar, 200 mm.
11. Cryopreserve the naive iPSC clones.
  a. Detach the naive iPSCs as described in the Steps 67 through 72.
  b. After centrifuging and discarding the supernatant, resuspend the cells with 1 mL of STEM-CELLBANKER.
  c. Count the cell number and prepare vials for cryopreservation (up to 5 × 10^6^ cells per vial).
  d. Add the STEM-CELLBANKER to the cell suspension according to the number of vials required, and aliquot the cell suspension to the vials.
  e. Put the cell-containing vials in a freezing container and store them at −80°C.
  f. Transfer and store the vials at LN2 temperatures (−169°C to −196°C) from the next day onward.

***Note:*** When re-starting the naive iPSC culture by thawing these vials, cell growth is often slow during the first 1–2 passages.

7. Reseed the SeV-infected HDFs on the iMEF plate (Day 5, HDF protocol only) (Timing: 1–2 h)
  a. Warm the fibroblast medium to 20°C–25°C.
    **CRITICAL:** Do not warm the medium to 37°C.
  b. Remove the medium from the well containing the SeV-infected cells.
  c. Wash the well with 2 mL of PBS.
  d. Add 1 mL of Accutase.
  e. Incubate at 35°C for 10 min.
    **CRITICAL:** Do not use a 37°C incubator to avoid inactivation of the SeV vectors.
  f. Dissociate the cells by pipetting and transfer the cell suspension into a 15 mL tube containing 5 mL of the fibroblast medium.
  g. Rinse the well by adding 1 mL of the fibroblast medium to the well.
  h. Add the suspension to the same 15 mL tube to collect the cells remaining in the well.
  i. Centrifuge at 500 × *g* for 5 min.
  j. Aspirate the supernatant.
  k. Resuspend the cells in 1 mL of the fibroblast medium.
  l. Count the cell number using an automated cell counter. Add the fibroblast medium to a concentration of 1.0 × 10^5^ cells/mL.
  m. Wash the iMEF plate with PBS.
  n. Plate the cell suspension on the iMEF plate (2.0 × 10^5^ cells/well).
8. Switch to the t2iLGö+Y medium (Day 6, HDF protocol only) (Timing: 20 min).
  a. Prepare the t2iLGö+Y medium and warm it to 20°C–25°C.
    **CRITICAL:** Do not warm the medium to 37°C.
  b. Remove the fibroblast medium from the well.
  c. Add 2 mL of the t2iLGö+Y medium.
  d. Incubate at 35°C, 5% CO_2_ and 5% O_2_.
  e. Change the medium every other day.
    ***Note:*** Typically, dome-shaped naive iPSC-like colonies with shiny edges appear around 10 days after the SeV infection. If colonies do not appear after 14 days, it is highly likely that the reprogramming was not successful.
9. Passage the naive iPSCs (around day 14) (Timing: 3 h).
  ***Note:*** Prepare the iMEF plate the day before passaging the naive iPSCs.
  ***Note:*** The timing for passaging is usually around 14 days after the SeV infection (around 4 days after the appearance of colonies), but if the naive iPSC colonies begin to detach, passage the cells at an earlier time.
  a. Prepare and warm the t2iLGö+Y medium to 20°C–25°C.
  b. Remove the medium from the well containing naive iPSCs.
  c. Wash the well with PBS.
  d. Add 1 mL of Accutase.
  e. Incubate at 35°C for 10 min.
  f. Dissociate the cells by pipetting and transfer the cell suspension into a 15 mL tube containing 5 mL of the wash medium.
  g. Centrifuge at 500 × *g* for 5 min.
  h. Aspirate the supernatant and resuspend the cells with 1 mL of the t2iLGö+Y medium.
  i. Wash the iMEF plate twice with PBS.
  j. Add 1.5 mL of the t2iLGö+Y medium.
  k. Plate 0.5 mL of the cell suspension on the iMEFs into the 6-well plate (total volume, 2 mL/well).
  l. Incubate at 35°C, 5% CO_2_ and 5% O_2_.
    ***Note:*** It is recommended to passage the naive iPSCs at a 1:2 ratio for the first 2–3 passages, because some cells disappear after raising the temperature. If the number of colonies is extremely low, consider passaging all of them, and if the number is obviously high, consider passaging 1/4 of the cells.
10. Change the temperature to remove the SeV vectors (Day 15-) (Timing: 5 min).
  a. Change the incubation temperature from 35°C to 38°C to remove the SeV vectors.
  b. Replace the medium, warmed to over 37°C, every other day.
    **CRITICAL:** Do not use unwarmed medium. Using cold medium slows down the removal of SeV.
  c. Passage cells every 3–4 days at a ratio of 1/4 of the original cell number.
  d. Around day 34 (after 20 days culturing at 38°C following the temperature change from 35°C), sample the naive iPSCs left over from the passage and extract the RNA using RNeasy Mini Kit according to the manufacturer’s protocol.
  e. Synthesize cDNA using PrimeScript RT Master Mix according to the manufacturer’s protocol.
  f. Perform qPCR using SeV TaqMan probes according to the manufacturer’s protocol to check for the presence of absence of residual SeV genomes.
  g. After confirming the removal of the SeV vectors, change the temperature to 37°C (Figure 3).
    **CRITICAL:** SeV vectors can be removed at 37°C as well, but 38°C is strongly recommended for faster SeV removal.
    ***Note:*** If cell proliferation is very slow, passage all the cells or half of the cells (1:2 ratio). Extending the passage period beyond 4 days is not recommended, as it often causes cell colony detachment.

### Feeder-free culture of the generated naive iPSCs (optional, HDF protocol only)

**Timing: 15 days**

This method has been confirmed to be effective only for HDF-derived naive iPSCs. Use a stable HDF-derived naive iPSC line that has been passaged several times after the naive reprogramming.

Typically, feeder-free conversion is considered successful if naive iPSCs continue to proliferate while maintaining their colony morphology for approximately four passages or more after the initiation of feeder-free culture.

12. iMEF-conditioned t2iLGö+Y medium preparation (Timing: 3 days).
  Before adapting naive iPSCs to a feeder-free culture, it is necessary to prepare iMEF-conditioned t2iLGö+Y medium.
  a. Seed iMEF (day 1) (Timing: 30 min).
    i. Warm the fibroblast medium to 37°C.
    ii. Add 0.1% gelatin solution to a 15 cm dish and incubate at 20°C–25°C for at least 15 min.
    iii. Take a frozen iMEF vial (4 × 10^6^ cells/vial) from the LN2 tank.
    iv. Thaw the frozen vial of iMEF in a 37°C water bath.
    v. Transfer the content of the vial into a 50 mL tube containing 19 mL of the fibroblast medium.
    vi. Aspirate the gelatin solution from the wells just before the cell plating.
    vii. Plate the cell suspension to the gelatin-coated 15 cm dish (2.5 × 10^4^ cells / cm^2^, e.g., a 4 × 10^6^ iMEF cell vial for a 15 cm dish with 20 mL of the fibroblast medium).
    viii. Incubate at 37°C with 5% CO_2_ for more than 12 h.
  b. Change medium to the t2iLGö medium (day 2) (Timing: 20 min).
    i. Prepare the t2iLGö medium and warm it to 20°C–25°C.
      ***Note:*** Do not add Y-27632 to the medium.
    ii. Aspirate the fibroblast medium and add 20 mL of the t2iLGö medium.
    iii. Incubate at 37°C with 5% CO_2_ for 24 h.
  c. Collect the iMEF-conditioned t2iLGö medium (day 3) (Timing: 30 min).
    i. Collect the medium
    ii. Filter the medium using 0.45 μm low-protein binding filter to remove floating cells and debris.
    iii. Aliquot the medium according to the amount used in cell culture and store at −20°C. Store at 4°C for up to 2 weeks after thawing.

**Pause point:** This medium can be stored at 4°C for 2 weeks or −20°C for up to 6 months.

**Figure 4:**
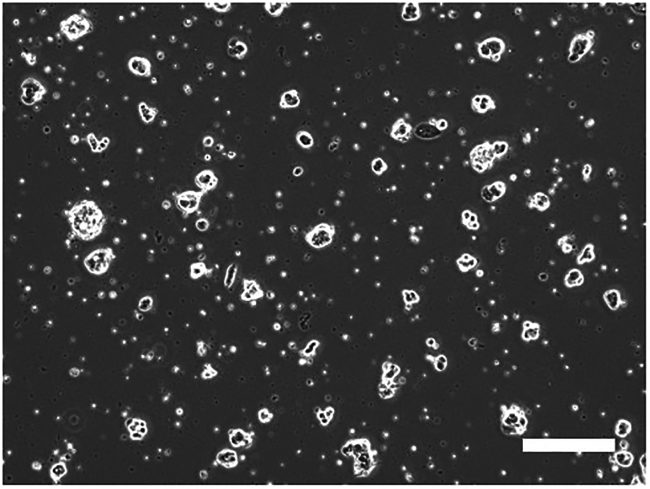
Feeder-free naive iPSC colonies derived from HDFs Representative phase-contrast images of naive iPSCs reprogrammed with Naive reprogramming cocktail (SeV-KOS, SeV-KLF4/TS12, and SeV-LMYC vectors) in feeder-free condition. Scale bar, 200 mm.

***Note:*** In the first few passages, all of the cells should be passaged because there will be low numbers of cells. However, if the cells are clearly increasing in number, the number of cells to be passaged can be reduced as appropriate.

***Note:*** Some cell lines differentiate suddenly around 4 passages, so carefully monitor the cells. If the naive-like morphology is maintained after the 4th passage, that cell line has a high probability of being stably maintained feeder-free thereafter.

13. Remove the iMEFs and passage the naive iPSCs (Timing: 4 h).
  ***Note:*** It is recommended to prepare an ample number of naive iPSCs in advance, as some naive iPSCs will also adhere to the plate and be lost during the two-hour incubation period.
  ***Note:*** Prepare the iMEF-conditioned t2iLGö+Y medium by adding 10 μM Y-27632 immediately before use.
  a. Prepare PBS-diluted Matrigel (4 μL Matrigel / cm^2^, e.g., 38.4 μl Matrigel with 1 mL PBS per well of a 6-well plate) and immediately coat a 6-well plate.
  b. Incubate at 37°C for 1 h.
  c. Add 0.1% gelatin solution to a second 6-well plate and incubate at 20°C–25°C for at least 15 min.
  d. Warm the iMEF-conditioned t2iLGö+Y medium to 20°C–25°C.
  e. Remove the medium from the well that contains naive iPSCs
  f. Wash the well with PBS.
  g. Add 1 mL of Accutase
  h. Incubate at 37°C for 10 min.
  i. Dissociate the cells by pipetting and transfer the cell suspension into a 15 mL tube containing 5 mL of the wash medium.
  j. Centrifuge at 500 × *g* for 5 min.
  k. Aspirate the supernatant and resuspend the cells with 2 mL of the iMEF-conditioned t2iLGö+Y medium.
  l. Aspirate the gelatin solution in the gelatin-coated dish just before plating the cells.
  m. Plate the cell suspension.
  n. Incubate at 37°C, 5% CO_2_ and 5% O_2_ for 2 h to attach only iMEF to gelatin on the bottom.
    **CRITICAL:** Do not move the plate until 2 h have passed.
  o. Aspirate Matrigel-containing PBS off the Matrigel-coated well.
  p. Gently collect only the supernatant that contains naive iPSCs from the gelatin-coated plate.
  q. Plate the supernatant in the Matrigel-coated well.
  r. Incubate at 37°C, 5% CO_2_ and 5% O_2_.
  s. Change the media every other day.
  t. Four days later at the next timing of iPSC passage, repeat the steps 15–31 to completely remove iMEFs.
    ***Note:*** Methods of selecting naive iPSCs using naive-specific markers and a cell sorter^4^ may also be effective.
14. 3rd and subsequent passages of the feeder-free naive iPSCs (Timing: 1 h).
  a. Prepare the Matrigel-coated plate as described in the steps 15 and 16.
  b. Warm the iMEF-conditioned t2iLGö+Y medium to 20°C–25°C.
  c. Remove the medium from the well that contains naive iPSCs.
  d. Wash the well with PBS.
  e. Add 1 mL of Accutase.
  f. Incubate at 37°C for 10 min.
  g. Dissociate the cells by pipetting and transfer the cell suspension into a 15 mL tube containing 5 mL of the wash medium.
  h. Centrifuge at 500 × *g* for 5 min.
  i. Aspirate the supernatant and resuspend the cells with 2 mL of the iMEF-conditioned t2iLGö+Y medium.
  j. Aspirate Matrigel-containing PBS off the Matrigel-coated well.
  k. Plate the cell suspension on the Matrigel-coated 6-well plate (2 mL/well).
  l. Incubate at 37°C, 5% CO_2_ and 5% O_2_ (Figure 4).

## Expected outcomes

This protocol describes the method for generating transgene-free naive human iPSCs from HDFs and PBMCs. Naive iPSC colonies appear around 10 days after infection with the SeV vectors. Typically, the reprogramming efficiency of the naive iPSCs using this method is 0.1%–0.5%.^1^ After changing the incubation temperature to 38°C, SeV vectors disappear and the SeV genome is not detected by qPCR by around 20 days after the temperature shift.

## Limitations

Successful generation of feeder-free naive iPSCs has only been confirmed in some HDF lines. In our experiments, HDFs that produced a higher number of naive iPSC colonies during their generation showed a higher success rate in feeder-free adaptation, suggesting that high-quality reprogramming is essential.

We observe variations in quality among established naive cell clones especially using only OSKL. Therefore, if you find significant variation in the reprogramming efficiency between clones or in the differentiation ability of the established naive iPSC clones, it is recommended to use *H1FOO-DD* vector with OSKL.

We have also succeeded in generating naive iPSCs using 5iLA^5^ medium or PXGLY^6^ medium instead of t2iLGö+Y medium only from HDFs according to this protocol.^2^ However, we have not evaluated the detailed functions of these iPSCs as naive iPSCs, such as their differentiation potency to extraembryonic lineage,^7^ so it is still unclear whether they have the same degree of naiveness as naive iPSCs generated using t2iLGö+Y introduced in this protocol.

## Troubleshooting

### Problem 1

(For HDF reprogramming) After the SeV vectors infection, many cells become round in shape and finally detach from the dish.

### Potential solution

The MOI of the SeV vectors is too high. In this case, it is recommended that the MOI of each vector of OSKL be lowered (e.g., MOI 3) (see, Step 3). If you are using the H1FOO-DD vector, the MOI for H1FOO-DD is recommended to be 0.1–0.3.

### Problem 2

The naive iPSC colonies do not appear or the reprogramming efficiency is very low.

### Potential solution

• Do not use naive iPSC medium that has been warmed to 37°C until naive iPSC colonies Appear (see, Step 3).
• Check that the cell density of the iMEFs is appropriate. If it is significantly lower than the indicated cell concentration, the reprogramming efficiency will decrease (see, Step 6).
• Use H1FOO-DD vector in addition to OSKL. The MOI for H1FOO-DD is recommended to be 0.1–0.3 (see, Step 3).
• Increase the MOI of each SeV vector up to an MOI of 10. Note that too high MOI results in cell toxicity and can cause cell death (see, Step 3).
• Regarding HDFs, use cells derived from donors as young as possible. HDFs tend to have higher reprogramming efficiency at younger ages.

### Problem 3

SeV vectors are not removed from the naive iPSCs as expected.

### Potential solution

• Check the actual temperature of the incubator. The removal efficiency of SeV vectors may be greatly reduced if the temperature is lower than 38°C^8^ (see, medium and incubator setup).
• Use warm media when changing the media or passaging the cells after naive iPSC colonies appear (see, Step 10).
• If the MOI of the SeV used is higher than the recommended value, SeV persistence can be prolonged (see, Step 3).

## Resource availability

### Lead contact

Further information and requests for resources and reagents should be directed to and will be fulfilled by the lead contact, Akira Kunitomi akira.kunitomi@gladstone.ucsf.edu.

### Technical contact

Questions about the technical specifics of performing the protocol should be directed to and will be answered by the technical contact, Akira Kunitomi akira.kunitomi@gladstone.ucsf.edu.

### Materials availability

These modified SeV vectors in this protocol are available from the lead contact.

### Data and code availability

This protocol does not include the generation of datasets or codes.

## Acknowledgments

We are grateful to ID Pharma Co., Ltd. for developing and providing the SeV vectors and K. Claiborn for critical reading of this manuscript. This work was supported by the Core Center for iPS Cell Research, 10.13039/100018627Research Center Network for Realization of Regenerative Medicine, 10.13039/100009619AMED under grant number JP21bm0104001; iPS Cell Research Fund; and 10.13039/501100001691JSPS KAKENHI under grant numbers 16K19429 and 18K15846. This work was also supported by funding from Mr. Hiroshi Mikitani, Mr. Marc Benioff, the 10.13039/100009856L. K. Whittier Foundation, the 10.13039/100009724Roddenberry Foundation, the 10.13039/100008072Gladstone Institutes, the 10.13039/100000050National Heart, Lung, and Blood Institute (NHLBI), and 10.13039/100000002National Institutes of Health (NIH) (U01-HL100406, U01-HL098179, R01-HL130533, and R01-HL135358). 10.13039/100008072Gladstone Institutes received support from 10.13039/100000097National Center for Research Resources grant RR18928-01.

## Author contributions

K.W. performed the experiments and analyzed the data. S.Y. supervised the project. A.K. designed and conceived this study, performed the experiments, and analyzed the data. K.W. and A.K. wrote the manuscript.

## Declaration of interests

A.K. is a co-inventor on a patent describing the method for this naive reprogramming method. S.Y. is a scientific advisor to iPS Academia Japan and Altos Labs without salary.
